# Rapid Geriatric Assessment Using Mobile App in Primary Care: Prevalence of Geriatric Syndromes and Review of Its Feasibility

**DOI:** 10.3389/fmed.2020.00261

**Published:** 2020-07-08

**Authors:** Reshma Aziz Merchant, Richard Jor Yeong Hui, Sing Cheer Kwek, Meena Sundram, Arthur Tay, Jerome Jayasundram, Matthew Zhixuan Chen, Shu Ee Ng, Li Feng Tan, John E. Morley

**Affiliations:** ^1^Division of Geriatric Medicine, Department of Medicine, National University Hospital, National University Health System, Singapore, Singapore; ^2^Department of Medicine, Yong Loo Lin School of Medicine, National University of Singapore, Singapore, Singapore; ^3^National University Polyclinics, National University Hospital System, Singapore, Singapore; ^4^Department of Electrical and Computer Engineering, National University of Singapore, Singapore, Singapore; ^5^Healthy Ageing Program, Alexandra Hospital, National University Health System, Singapore, Singapore; ^6^Division of Geriatric Medicine, Saint Louis University School of Medicine, St. Louis, MO, United States

**Keywords:** geriatric syndrome, primary care, rapid geriatric assessment, iPad application, older adult, frailty, sarcopenia, anorexia of aging

## Abstract

With the aging population and consequent increase in associated prevalence of frailty, dementia, and multimorbidity, primary care physicians will be overwhelmed with the complexity of the psychosocial and clinical presentation. Geriatric syndromes including frailty, sarcopenia, cognitive impairment, and anorexia of aging (AA) either in isolation or in combination are associated with an increased risk of adverse outcomes and if recognized early, and appropriately managed, will lead to decreased disability. Primary care practices are often located in residential settings and are in an ideal position to incorporate preventive screening and geriatric assessment with personalized management. However, primary care physicians lack the time, multidisciplinary resources, or skills to conduct geriatric assessment, and the limited number of geriatricians worldwide further complicates the matter. There is no one effective strategy to implement geriatric assessment in primary care which is rapid, cost-effective, and do not require geriatricians. Rapid Geriatric Assessment (RGA) takes <5 min to complete. It screens for frailty, sarcopenia, AA, and cognition with assisted management pathway without the need of a geriatrician. We developed RGA iPad application for screening with assisted management in two primary care practices and explored the feasibility and overall prevalence of frailty, sarcopenia, and AA. The assessment was conducted by trained nurses and coordinators. Among 2,589 older patients ≥65 years old, the prevalence of frailty was 5.9%, pre-frail 31.2%, and robust 62.9%. Fatigue was present in 17.8%, and among them, the prevalence of undiagnosed depression as assessed by the Patient Health Questionnaire (PHQ)-9 was 76.4% and 13.5% of total. The prevalence of sarcopenia was 15.4%, and 13.9% experienced at least one fall in the past year. AA was prevalent in 10.9%. The time taken to do the assessment with defined algorithm was on average 5 min or less per patient, and 96% managed to complete the assessment prior to seeing their doctor in the same session. The RGA app is a rapid and feasible tool to be used by any healthcare professional in primary care for identification of geriatric syndrome with assisted management.

## Introduction

With the aging population and consequent increase in associated prevalence of frailty, dementia, and multimorbidity, primary care physicians will be overwhelmed with the complexity of the psychosocial and clinical presentation. There is evidence that geriatric syndromes, e.g., frailty, sarcopenia, falls, polypharmacy, anorexia/weight loss, cognitive impairment, and depression, if recognized early and appropriately managed will lead to decreased disability and mortality with better quality of life in older persons (1, 2). The World Health Organization Integrated Care for Older People guidelines recommends assessing older persons for declining physical and mental capacities with necessary interventions (3). Comprehensive geriatric assessment (CGA), initially introduced by Marjory Warren, extends beyond traditional medical history and incorporates an interdisciplinary diagnostic process to identify medical, functional, and psychosocial issues in order to develop a personalized care plan to maximize the well-being of the older adult (4, 5). The effectiveness of the CGA and evaluation was further supported by a meta-analysis which showed reduced hospitalization, death, and institutionalization (6). Geriatric assessment is often conducted by geriatricians or geriatric nurse practitioners and can take between 20 and 45 min (7). In the current acute-care centric setting and disease model of care, proactive screening for geriatric syndromes and case finding is done on *ad hoc* basis, and in many instances, it is not followed up with intervention (8, 9). While CGA is effective in hospitalized older adults presenting with falls, fracture, functional decline, and delirium and with shortage of geriatricians worldwide, preventive screening and geriatric assessment in primary care are the best possible solutions in the provision of upstream goal-directed person-centered care (7, 10, 11).

Primary care is the foundation of healthcare system in Singapore. More than 20 subsidized polyclinics and 1,700 general practice clinics are located island-wide in the residential setting and are in ideal position to do CGA with the necessary interventions (12). They are often the first line of contact and treat a whole range of conditions from upper respiratory tract infections to chronic diseases and keep the population healthy through preventive population health. The concept of teamlet was introduced in 2014 in the polyclinic where patient-centered care is provided for those with chronic diseases by a team of doctors, care coordinators, and supported by in-house allied healthcare staff (13). The teamlet focuses on patients' medical, functional, and psychological needs and provides holistic integrated care within the primary care setting. The focus has largely been on chronic diseases, and they work under constraints of limited time and limited multidisciplinary resources and may not have the necessary skills to conduct CGA. While not a problem locally, reimbursement issues in certain countries may be an obstacle for them to implement CGA as a routine practice. There is no one effective strategy to implement CGA in primary care which is rapid and cost-effective and does not require geriatricians or geriatric trained nurse clinicians to perform the assessments (10, 14). Many brief screening tools have been developed worldwide to be used in primary care including Gerontopôle Frailty Screening Tool (GFST) which includes a dedicated pathway for disability prevention, Kihon Checklist (KCL) which is a self-reported comprehensive health checklist, Vulnerable Elders Survey-13 (VES-13) which is a self-administered questionnaire with the aim of identifying those at risk of death or functional decline, Easycare Two-step Older persons Screening (Easycare-TOS) which is a brief standardized tool for assessing perceptions of older adults about their health and care needs, and the Electronic Frailty Index in the United Kingdom (11, 15, 16). Most assessment tools are predominantly screening or case finding tools with limited or no interventions recommended.

Rapid Geriatric Assessment (RGA) is one of the most practical tools developed at St. Louis University and takes <5 min to screen for frailty, sarcopenia, anorexia of aging (AA), and cognition and does not require a geriatrician to administer the assessment (1, 16). RGA comprises of four screening tools including the FRAIL scale for frailty, the SARC-F scale for sarcopenia, the Simplified Nutritional Appetite Questionnaire (SNAQ) for AA, and the Rapid Cognitive Screen (RCS) for cognition (17) (Supplementary 1).

The aim of this study was to explore the feasibility and implementation of RGA iPad application in two busy primary care practices and to determine overall prevalence of frailty, sarcopenia, and AA.

## Materials and Methods

**Figure 1:**
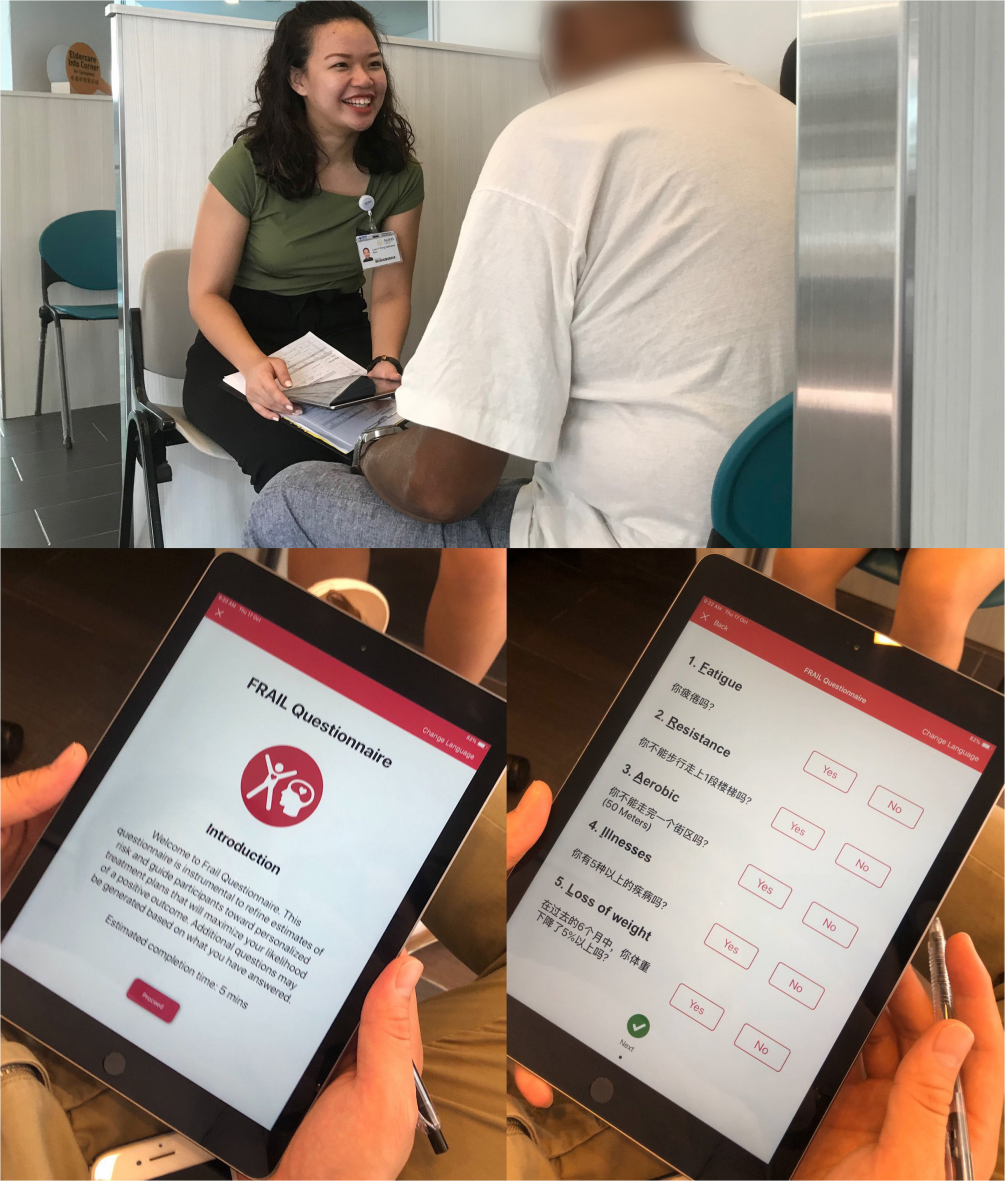
RGA mobile iPad app and screening.

An iPad mobile application for RGA was developed in English and Chinese, and screening was carried out in two primary care practice teamlets in the Western region of Singapore from April 2019 to December 2019 (Figure 1). Screening was done by trained care coordinators and/or nurse for 2,710 older patients ≥65 years old, and 2,589 had complete data collected. The assessment was carried out for those who had appointments to see their doctor on the same day. There are no copyright issues with RGA as the questionnaires belong to John E. Morley.

**Figure 2:**
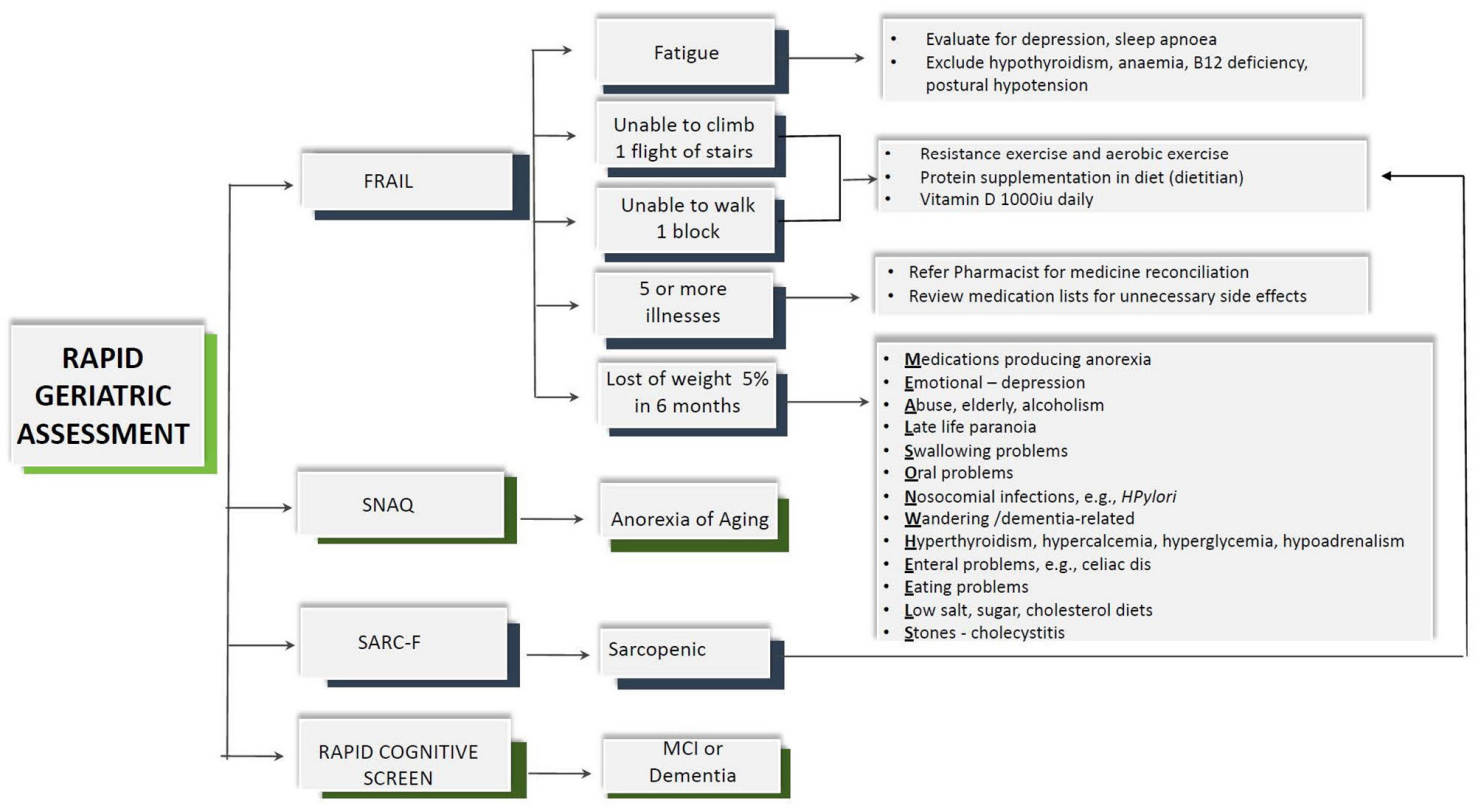
Rapid geriatric assessment and personalized management.

As part of the RGA, frailty was screened using the FRAIL scale with dedicated care pathway, sarcopenia using SARC-F, nutrition status using the SNAQ, and cognition using the RCS (Figure 2) (18). The assessment tool in RGA has been validated in many different countries and clinical conditions and has been shown to predict adverse outcomes such as mortality, functional decline, falls, and hospitalization (19). The five-item FRAIL scale (Fatigue, Resistance, Aerobic, Illness, and Loss of Weight) screens for frailty. The scores range from 0 to 5, where scores of 1–2 are considered pre-frail and 3–5 represent frail. The SARC-F screens for sarcopenia and comprises of five questions including strength, rise from a chair, assistance with walking, climbing stairs, and falls in the past year. A total score of ≥4 indicates sarcopenia. SARC-F has sensitivity ranging from 25 to 50% and specificity ranging from 90 to 98% (20, 21). The SNAQ includes four questions on appetite, taste of food, portion consumed, early satiety, and number of meals consumed daily. The total SNAQ score ranged from 4 to 20, and score of ≤ 14 predicts at least 5% of weight loss within 6 months with a sensitivity of 81.5% and a specificity of 76.4% (22). RCS includes four questions to screen for mild cognitive impairment or dementia. It includes five-item recall, clock drawing, and story recall. A total score of ≤ 5 indicates dementia, 6–7 mild cognitive impairment, and 8–10 indicates normal cognition (23).

The assisted management pathway algorithm is shown in Figure 2. For those who screened positive for fatigue, additional questions included screening for sleep apnea and depression. Depression was assessed using the Patient Health Questionnaire (PHQ-9) which is a self-administered version of the PRIME-MD diagnostic instrument for common mental disorders. It consists of nine items, and each item is assessed on a four-point scale (0 = not at all, 1 = several days, 2 = more than half the days, 3 = nearly every day) with scores ranging from 0 to 27 (24). PHQ-9 has been locally validated in Asian primary care setting (25).

While most information on the iPad app was self-explanatory, the coordinators and nurses were trained in administering the RGA. There was no prior CGA carried out in the primary care setting. Feasibility and practicality were measured using the average time taken to complete the assessment including the algorithm and the completeness of the assessment prior to seeing their doctor on the same day. There was no additional time allocated nor were the patients advised to come early for the assessment. The patients were given a piece of paper incorporating the assessment findings and scores to be handed over to their doctor on the same day. Outcome and recommended interventions of RGA were not evaluated.

Data were analyzed using SPSS Version 25.0 (IBM Corp., 2017). Characteristics of participants were presented as mean, standard deviation for continuous variables, while categorical variables were presented as frequencies (percentages). Differences between genders were assessed using two-sample *t*-test (when normality and homogeneity assumptions were satisfied); otherwise, Mann–Whitney *U*-test was used for continuous variables, while chi-square test was used for categorical variables. Statistical significance was set at *p*-value of < 0.05.

As this was performed as part of the routine clinical care in the teamlets within primary care, no ethics approval was necessary. The ethics approval was obtained, and consent was required if the older patients who were identified as pre-frail or frail were interested to participate in a subsequent intervention study.

## Results

Among the 2,589 older patients, 1,358 (52.5%) were women, and overall mean age was 73.1 years (Table 1). For living arrangements, almost two thirds were still living with family, with only 116 (8.5%) women and 77 (6.3%) men living alone. Among them, 579 (22.4%) were still working with only 13.2% in full-time employment. The overall prevalence of frailty was 5.9%, pre-frail 31.2%, and robust 62.9%. There was a significant difference between men and women where 6% of women compared with 4.2% of men were frail. The prevalence of sarcopenia was 15.4, and 20.6% of women compared with 9.6% of men were assessed to be sarcopenic. AA was prevalent in 283 (10.9%) older patients with prevalence in women being almost double that of men. RCS has not been validated locally, and hence the data for cognition is only available for 190 older patients. One third of the older patients screened had evidence of underlying cognitive impairment.

**Table 1:** Demographics of participants.

	**Overall** **N = 2,589**	**Men** **1,226 (47.4)**	**Women** **1,358 (52.5)**	***P*-value**
Age (mean, SD)	73.1, 6.5	72.8, 6.2	73.3, 6.7	**0.030**
Living arrangement				**<0.001**
Alone	193 (7.5)	77 (6.3)	116 (8.5)	
Spouse	699 (27.0)	423 (34.6)	275 (20.3)	
Family (Spouse/Children/ Others/Friend)	1,651 (63.7)	716 (58.4)	930 (68.5)	
Domestic Helper	30 (1.2)	4 (0.3)	26 (1.9)	
Landlord/Tenant	17 (0.7)	6 (0.5)	11 (0.8)	
Employment				**<0.001**
Retired	1,042 (40.2)	789 (64.4)	558 (41.1)	
Full time	341 (13.2)	250 (20.4)	186 (13.7)	
Part time	238 (9.2)	124 (10.1)	115 (8.5)	
Home maker/housewife	821 (31.7)	24 (2.0)	418 (30.8)	
Unemployed	148 (5.7)	39 (3.2)	81 (6.0)	
Smoking				**<0.001**
Never smoked	2,090 (80.7)	770 (62.8)	1,315 (96.8)	
Current smoker	164 (6.3)	144 (11.7)	19 (1.4)	
Past smoker	336 (13.0)	312 (25.4)	24 (1.8)	
Frailty Status				**<0.001**
Robust	1,628 (62.9)	821 (67.0)	818 (60.2)	
Pre-frail	809 (31.2)	349 (28.5)	459 (33.8)	
Frail	151 (5.9)	56 (4.1)	81 (6.0)	
Sarcopenia	399 (15.4)	118 (9.6)	280 (20.6)	**<0.001**
Anorexia of Aging	283 (10.9)	96 (7.8)	185 (13.6)	**<0.001**
Cognitive Impairment^1^	190	97 (51.1)	92 (48.9)	0.060
Normal	125 (65.8)	70 (72.2)	55 (59.8)	
MCI	26 (13.7)	14 (14.4)	12 (13.0)	
Dementia	39 (20.5)	13 (13.4)	26 (27.2)	

Values are n (%) unless otherwise noted.

1 *Rapid Cognitive Screen was conducted in 190 participants; percentages are of remaining men and women participants. Bold implies significance*.

**Figure 3:**
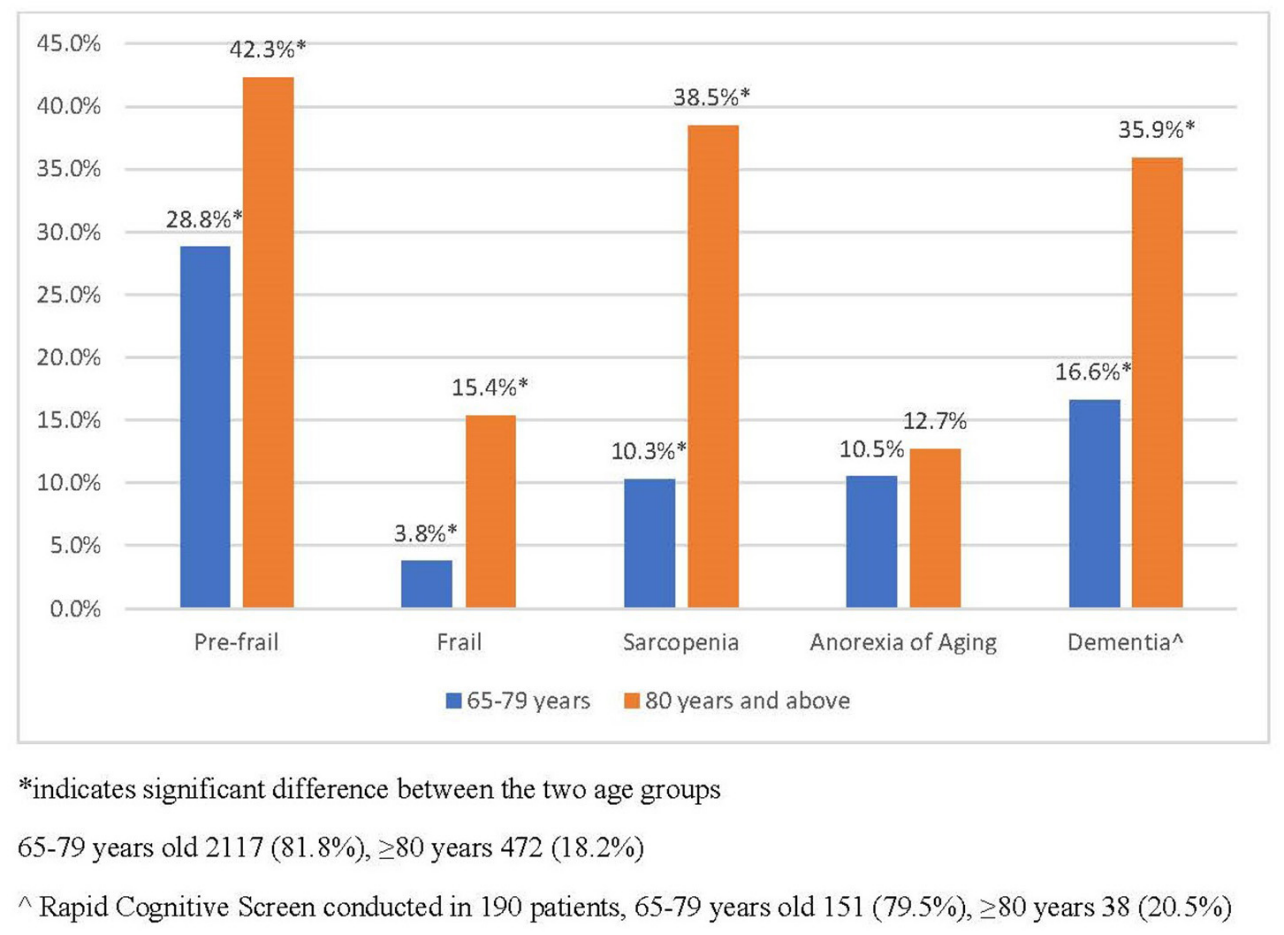
Prevalence of geriatric syndromes in the old and old–old in primary care. *indicated significant between the two age groups. 65–79 years old 2,117 (81.8%), ≥80 years 472 (18.2%). ^∧^Rapid cognitive screen conducted in 190 patients, 65–79 years old 151 (79.5%), ≥80 years 38 (20.5%).

Figure 3 is the breakdown of geriatric syndromes among the old (65–79 years old) and old-old (≥80 years old). Prevalence of frailty among the old-old was 15.4%, which is almost five times more compared with 3.8% of the young old. More than one in three of the old-old were sarcopenic compared with one in 10 of the old group. Prevalence of dementia as assessed using the RCS was 35.9% among the old-old.

FRAIL has five items (Fatigue, Resistance, Aerobic, Illness, and Loss of Weight) and as shown in Figure 2, each item has personalized care management pathway. Fatigue was present in 17.8%:15.4% of men and 20% of women (Table 2A). Among those who were fatigued, the prevalence of sleep apnea was 4.8%. Depression as assessed by the PHQ-9 was prevalent in 352 (76.4%) older patients who were feeling fatigued and 13.6% of total. Almost half had minimal depression, and one in 10 had moderate to severe depression which had not been diagnosed prior to the implementation of RGA. One in seven older patients was unable to climb one flight of stairs, and similar numbers were also unable to walk one bus-stop distance which is about 50 m. Overall, 252 (9.8%) older patients had five or more chronic illnesses, which they were treated for, with only 7.9% of women compared with 11.8% of men. Loss of 5% or more weight in the last 6 months was reported by 3.6% of men and 4.6% of women. Among those with weight loss, additional questions on eating problems found that 8.3% reported issues with choking or coughing on eating, and 6.5% have issues with chewing or lack of teeth (Supplementary 2).

**Table 2A:** Frailty and subcomponents.

**Frailty**	**Overall** **2,589**	**Men** **1,226 (47.4)**	**Women** **1,358 (52.5)**	***P*-value**
Fatigue	461 (17.8)	189 (15.4)	272 (20.0)	**0.003**
Sleep apnea^*^	21 (4.8)	11 (5.8)	10 (3.6)	**0.007**
Depression^*^	352 (76.4)	126 (66.7)	226 (83.1)	0.199
Minimal depression	192 (48.0)	69 (45.7)	123 (49.4)	
Mild depression	119 (29.8)	45 (29.8)	74 (29.7)	
Moderate to severe depression	41 (10.3)	12 (7.9)	29 (11.6)	
Unable to climb 1 flight of stairs	351 (13.6)	103 (8.4)	248 (18.2)	**<0.001**
Unable to walk 1 Bus Stop	358 (13.8)	126 (11.8)	232 (17.1)	**<0.001**
Five or more chronic illnesses	252 (9.8)	145 (11.8)	107 (7.9)	**0.001**
Loss of weight	107 (4.1)	44 (3.6)	63 (4.6)	0.181

Values are n (%) unless otherwise noted.

* *Percentages are of remaining men and women participants who complained of fatigue. Bold implies significance*.

Sarcopenia was assessed using SARC-F comprising of five questions including strength, rise from a chair, assistance with walking, climbing stairs, and falls in the past year. Overall, one in four older patients had difficulties carrying 4.5 kg of weight, with one in three women and one in six men reporting difficulties (Table 2B). More women (17.3%) compared with men (9.9%) reported difficulties walking across the room. More than one in four had difficulties transferring from chair to bed, and similar numbers also had difficulties climbing one flight of stairs, with prevalence being much higher in women; 33.2% had difficulty transferring from a chair or bed, and 31.9% had difficulty climbing a flight of 10 stairs. The overall prevalence of one or more falls was 13.9% affecting 142 (11.6%) of men and 218 (16.1%) women.

**Table 2B:** Sarcopenia and subcomponents.

**SARC-F**	**Overall** **2,589**	**Men** **1,226 (47.4)**	**Women** **1,358 (52.5)**	***P*-value**
Sarcopenia	399 (15.4)	118 (9.6)	280 (20.6)	<0.001
Difficulty lifting and carrying 4.5 kg	658 (25.4)	199 (16.2)	458 (33.7)	<0.001
Difficulty walking across a room	357 (13.8)	121 (9.9)	236 (17.3)	<0.001
Difficulty transferring from a chair or bed	705 (27.2)	252 (20.6)	452 (33.2)	<0.001
Difficulty climbing a flight of ten stairs	663 (25.6)	229 (18.7)	433 (31.9)	<0.001
Falls in the last year	361 (13.9)	142 (11.6)	218 (16.1)	0.005

*Values are n (%) unless otherwise noted*.

The time taken to do the assessment and generate the above assessment was on average 5 min or less per patient as time was shown on the iPad screen. Time taken to complete was evaluated both by the authors for their own clinic patients before rolling out officially, and feedback was obtained from care coordinators and nurses. Not all patients were required to complete the full algorithm. As there was no additional time allocated, and patients usually came on time for their appointment, 2,710 patients were assessed, and full assessment was available for 2,589 patients. The rest could not complete assessments as they were called in by their doctors. Overall, 96% managed to complete the assessment prior to seeing their doctor.

## Discussion

Geriatric syndromes including frailty, sarcopenia, cognitive impairment, and AA either in isolation or combination are associated with increased risk of adverse outcomes including hospitalization, falls, functional decline, institutionalization, and mortality (26–29). Current healthcare is based on traditional disease-based model which is no longer sustainable in countries with a fast aging population where rise in multimorbidity and associated consequences will require proactive, upstream, person-centered care, multidisciplinary primary care approach to early identification of geriatric syndrome, assessment, and management (30).

Frailty is characterized by diminished strength and endurance due to accumulation of multiple deficits and known to be due to loss of harmonic interactions between different dimensions including psychological, socioeconomic, genetic, biological, and functional domains contributing to higher adverse outcomes (31). It is a state of gradual functional decline over 5–10 years eventually leading to disability during which there are many opportunities for early case finding and intervention before the onset of disability. The prevalence of frailty increases with age, and half of frail older adults are still independent (32, 33). Sarcopenia is an age-related loss of strength, skeletal muscle mass, and function. SARC-F is the recommended screening tool for sarcopenia in many international guidelines and has been validated in many countries worldwide and has a good predictive value for future dependency (34–36). Nutrition, frailty, and sarcopenia are closely related, and a potentially modifiable factor for prevention or delaying onset of frailty and sarcopenia. AA is a precursor for geriatric syndromes and malnutrition. The SNAQ has been validated worldwide in different diseases and settings to identify those at risk of malnutrition. The concurrent assessment for frailty, sarcopenia, AA, and cognition is important for upstream interventions even before the onset of geriatric syndromes. The above conditions share similar risk factors which are reversible with early intervention including polypharmacy, low physical activity, nutrition, chronic diseases including diabetes, depressive symptoms, poor social network, and undiagnosed cognitive impairment (37–40).

While it may be preferred for screening tools in primary care to have high sensitivity for the diagnosis of frailty and other geriatric syndromes, screening tools in RGA incorporating assisted management pathway provide not just diagnosis but intervention even before the onset of geriatric syndromes which makes it unique. A person with falls identified with SARC-F and at risk of AA may not screen positive for sarcopenia or frailty, but early intervention and assessment may delay the onset of sarcopenia and frailty. Sarcopenia can lead to physical frailty, causing poor balance, low muscle strength, falls, and fracture.

Primary care is the core component of healthcare where preventive healthcare is most feasible due to the accessibility and location within the community setting. In addition to assessing for frailty, the British Geriatric Society (BGS) recommends referral to the geriatric team for CGA (41). Due to limited numbers of geriatricians and the fact that frailty, sarcopenia, AA, and cognitive impairment are often difficult to diagnose in earlier stages, a fast and practical tool is needed to facilitate case finding in primary care (42). Primary care practices worldwide are often overwhelmed with multitudes of clinical issues ranging from neonates to frail older adults, and CGA is often not on their cards as it is time-consuming. Despite advances worldwide in developing practical tools in primary care, the overall take-up has been low due to multiple reasons including lack of skills, time, and reimbursement (9, 16). In addition, in countries like Singapore where we have different ethnic groups, there's often a language barrier in conducting CGA. To address these issues, RGA app was developed in English and Chinese. In addition, primary care physicians were not required to do CGA or RGA; they were just told to manage relevant positive findings.

Fatigue in the FRAIL scale is a well-known presenting complaint for those who are depressed, suffering from sleep apnea, or have underlying medical conditions such as anemia, hypothyroidism, hypotension, and B12 deficiency. Fatigue, depression, and B12 deficiency also accelerates cognitive decline. The “R” and “A” component of FRAIL and sarcopenia can be managed with resistance exercise and a high-protein diet. The “I” refers to more than five illnesses which can be managed by reviewing medications and reducing inappropriate prescribing. Loss of weight and/or AA has many treatable factors (Figure 2). In addition, SARC-F also has a question on falls, and if screened positive can be referred for additional evaluation which includes vision.

The prevalence of frailty in primary care was 5.1% which is very similar to community prevalence (32). In addition to prevalence of frailty, among those who were fatigued, the prevalence of undiagnosed depression was 76.4%, which makes it 13.6% overall. While the number is significant, the prevalence is much lower than that in other studies where it ranged from 29.9 to 37.2% (43, 44). However, the prevalence in our study is similar to other studies done locally reporting the prevalence between 3.7 and 13.3% (25, 45, 46). Depression in older adults in primary care often goes unrecognized and carries a poor prognosis, and RGA made it possible to screen for depression using a simple question of fatigue where almost three quarters were screened to have depression (47). Sleep apnea has been associated with fatigue, cognitive impairment, falls, and functional impairment (48). The overall self-reported prevalence in our patients was 4.8% which is much lower than the prevalence reported using polygraphy in the population-based cohort study not restricted to older adults (49, 50). The prevalence of sleep apnea increases with age and often under-recognized by patients and caregivers (51).

The prevalence of sarcopenia in our older patients in primary care was 15.4%, significantly higher in women. More importantly, RGA was able to identify those with difficulties transferring and climbing one flight of stairs and those who suffered a fall. As there is no gold standard for diagnosis of sarcopenia, the prevalence within community varies from 4 to 23% (52).

Malnutrition and AA are often used interchangeably, when in fact one is a precursor of the other and early interventions may prevent malnutrition, weight loss, and cachexia (53–55). Slightly more than half of older adults at risk of malnutrition are known to be either frail or pre-frail (56). The prevalence of AA ranges between 10.7 and 25% in community-dwelling older adults (57, 58). Despite its high prevalence and significance, it is often overlooked by healthcare professionals and attributed to normal aging. The prevalence of AA in our older patients in primary care was 10.9%, being significantly higher in women, which is very similar to the Japanese (58). Only 4.1% of the older patients reported significant weight loss.

The RGA app was not intended to replace assessment by primary care clinicians but to enhance and enable case finding among those at risk. There were initial concerns among primary care doctors on consultation time if additional issues needed addressing. While the additional issues were flagged up to managing primary care physician, we do not have data on interventions offered and outcome including the development of geriatric syndrome and/or healthcare utilization. In addition, we do not have data on behavior change or knowledge on geriatric syndromes among primary care physicians. Longitudinal follow-up of these patients will be required. However, the process was seamless, and it was eventually accepted as usual practice. The RGA app is unique compared to other geriatric assessment tools developed for primary care in that it includes personalized management assistance pathway and can be administered by any health care professional and/or coordinator. The time taken was evaluated initially before and just after rolling out, and feedback was obtained from the team subsequently. It was both feasible, requiring on average 5 min or less per patient, and practical where it did not require additional resources, and complete data were obtained for 96% of the patients assessed. RGA with management assistance iPad application is rapid and practical and can be used in any primary care practice for identification and management of geriatric syndromes without the need of additional time or resources.

## Conclusion

The RGA app is a rapid and feasible tool to be used in primary care for identification of geriatric syndrome and assisted management. It can be used by any healthcare professional to identify the at-risk population. Psychosocial aspects especially loneliness can be incorporated in the future including the use of artificial intelligence to further fine-tune the identification of at-risk seniors (59).

## Data Availability Statement

The datasets for this article are not publicly available as this is more of a feasibility study using an app to enroll participants in intervention study. Requests to access the datasets should be directed to Reshma Aziz Merchant, reshmaa@nuhs.edu.sg.

## Ethics Statement

The studies involving human participants were reviewed and approved by NHG DSRB (National Healthcare Group Domain Specific Review Board). The written informed consent wasn't obtained for this study as Rapid Geriatric Assessment was done as a routine care protocol in primary care, and willingness to answer the questions was deemed as an informed consent. Written informed consent was obtained from the participants of the study (as required by the ethics committee) once they were enrolled in any intervention after screening, and written informed consent was obtained from the individuals for the publication of any potentially identifiable images or data included in this article.

## Author Contributions

RM, RH, SK, MS, AT, JJ, MC, SN, LT, and JM contributed to conceptualization. RM, RH, and SK contributed to the formal analysis. RM, MC, and LT contributed to funding acquisition. RM, AT, JJ, and JM contributed to app design and creation. RM, RH, SK, MS, and JM contributed to methodology and project administration. RM contributed to writing the original draft. RH, SK, MS, AT, JJ, MC, SN, LT, and JM contributed to review and editing. All authors contributed to the article and approved the submitted version.

## Conflict of Interest

The authors declare that the research was conducted in the absence of any commercial or financial relationships that could be construed as a potential conflict of interest.
