# Supplementary material for: Rapid Geriatric Assessment Using Mobile App in Primary Care: Prevalence of Geriatric Syndromes and Review of Its Feasibility
Source: Front Med (Lausanne). 2020 Jul 8;7:261. doi: 10.3389/fmed.2020.00261 (PMC7360669; doi:10.3389/fmed.2020.00261)
Supplement: Supplementary file 1 [file Data_Sheet_1.PDF]

Supplementary 1: Rapid Geriatric Assessment Tools and Scoring

Saint Louis University
Rapid Geriatric Assessment*

*There is no copyright on these screening tools and they may be incorporated into the Electronic Health Record without permission and at no cost.

ID#:____________Sex:____________Age:________Primary Care Provider Y / N

Ethnicity (circle): African/Am Asian Caucasian Hispanic Non-Hispanic

The Simple "FRAIL" Questionnaire Screening Tool

Fatigue: Are you fatigued?
Resistance: Cannot walk up one flight of stairs?
Aerobic: Cannot walk one block?
Illnesses: Do you have more than 5 illnesses?
Loss of weight: Have you lost more than 5% of your weight in the last 6 months?

Scoring: 3 or greater = frailty; 1 or 2 = prefrail

Total FRAIL Score: ____________

SARC-F Screen for Sarcopenia (Loss of Muscle)

Component	Question
Strength	How much difficulty do you have in lifting and carrying 10 pounds?
Scoring: None = 0 Some = 1 A lot or unable = 2	
Assistance in Walking	How much difficulty do you have walking across a room?
Scoring: None = 0 Some = 1 A lot , use aids or unable = 2	
Rise from a Chair	How much difficulty do you have transferring from a chair or bed?
Scoring: None = 0 Some = 1 A lot or unable without help = 2	
Climb stairs	How much difficulty do you have climbing a flight of ten stairs?
Scoring: None = 0 Some = 1 A lot or unable = 2	
Falls	How many times have you fallen in the last year?
Scoring: None = 0 1-3 Falls = 1 4 or more falls = 2	

Total score of 4 or more indicates Sarcopenia

Total SARC-F Score: ___________

SNAQ (Simplified Nutritional Assessment Questionnaire)

My appetite is
a. very poor
b. poor
c. average
d. good
e. very good

Food tastes
a. very bad
b. bad
c. average
d. good
e. very good

When I eat
a. I feel full after eating only a few mouthfuls
b. I feel full after eating about a third of a meal
c. I feel full after eating over half a meal
d. I feel full after eating most of the meal
e. I hardly ever feel full

Normally I eat
a. Less than one meal a day
b. One meal a day
c. Two meals a day
d. Three meals a day
e. More than three meals a day

Scoring: a=1, b=2, c=3, d=4, e=5.
A score, <14 indicates significant risk of at least 5% weight loss within 6 months.

Total SNAQ Score: ___________

Rapid Cognitive Screen (RCS)

1. **Please remember these five objects. I will ask you what they are later.**
   [Read each object to patient using approx. 1 second intervals.]
   Apple Pen Tie House Car
2. [Give patient pencil and the blank sheet with clock face.] **This is a clock face. Please put in the hour markers and the time at ten minutes to eleven o'clock**
   . [2 pts/hr markers ok; 2 pts/time correct]
3. **What were the five objects I asked you to remember?**
   [1 pt/ea]
4. **I'm going to tell you a story. Please listen carefully because afterwards, I'm going to ask you about it.**

Jill was a very successful stockbroker. She made a lot of money on the stock market. She then met Jack, a devastatingly handsome man. She married him and had three children. They lived in Chicago. She then topped work and stayed at home to bring up her children. When they were teenagers, she went back to work. She and Jack lived happily ever after.
What state did she live in? [1 pt]

SCORING
8-10……... Normal
6-7………. Mild Cognitive Impairment
0-5………. Dementia

Total RCS Score: ___________

Permission obtained from Professor John E Morley
