# Supplementary figures and images for: Rapid Geriatric Assessment Using Mobile App in Primary Care: Prevalence of Geriatric Syndromes and Review of Its Feasibility
Source: Front Med (Lausanne). 2020 Jul 8;7:261. doi: 10.3389/fmed.2020.00261 (PMC7360669; doi:10.3389/fmed.2020.00261)

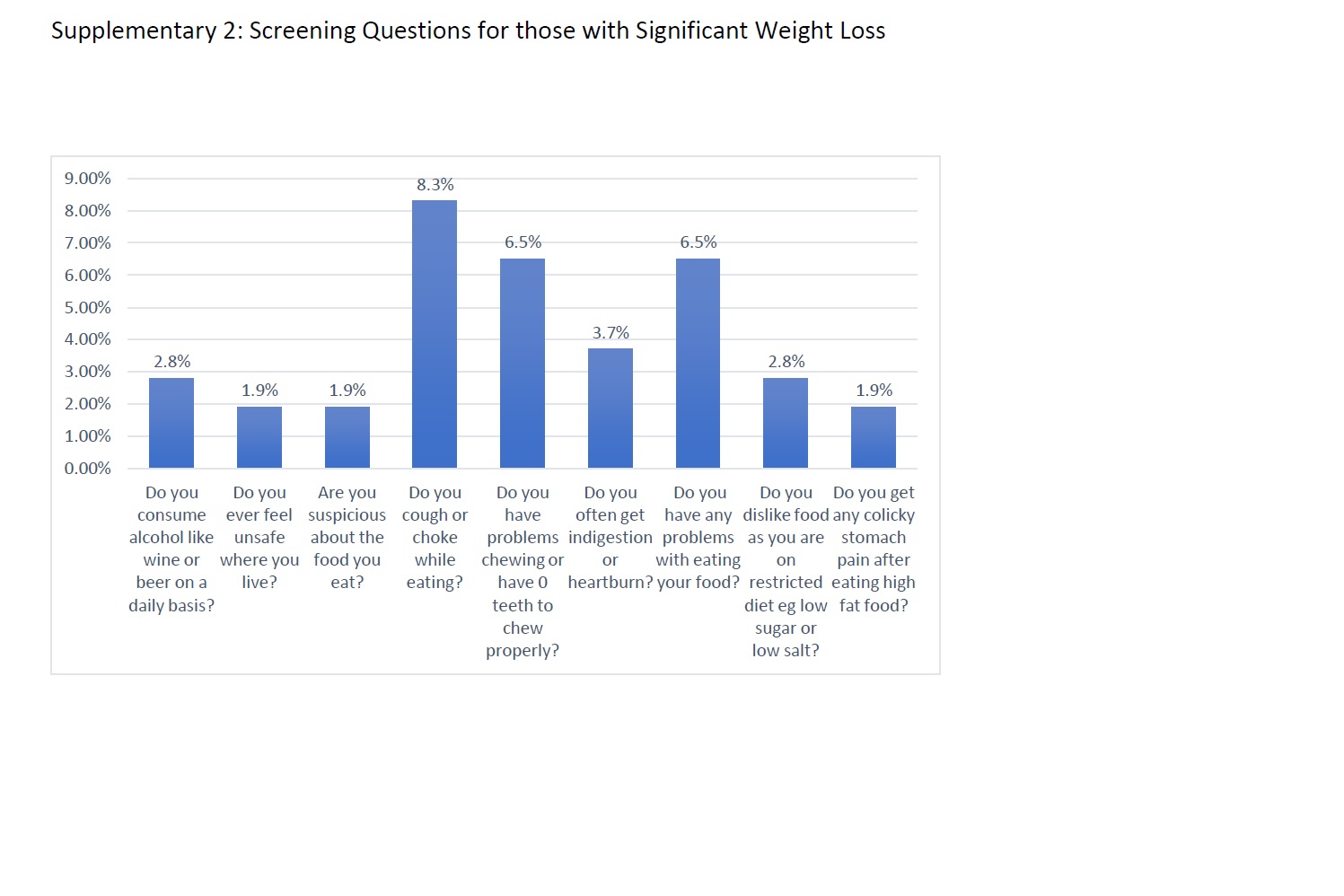

Supplement: Supplementary file 2 [file Image_1.JPEG]
